# Plant Ontology (PO): a Controlled Vocabulary of Plant Structures and Growth Stages

**DOI:** 10.1002/cfg.496

**Published:** 2005

**Authors:** Pankaj Jaiswal, Shulamit Avraham, Katica Ilic, Elizabeth A. Kellogg, Susan McCouch, Anuradha Pujar, Leonore Reiser, Seung Y. Rhee, Martin M. Sachs, Mary Schaeffer, Lincoln Stein, Peter Stevens, Leszek Vincent, Doreen Ware, Felipe Zapata

**Affiliations:** 1 Department of Plant Breeding 240 Emerson Hall Cornell University Ithaca NY 14853 USA; 2 Cold Spring Harbor Laboratory 1 Bungtown Road Cold Spring Harbor NY 11724 USA; 3 Department of Plant Biology Carnegie Institution of Washington 260 Panama Street Stanford CA 94305 USA; 4 Department of Biology University of Missouri at St. Louis St. Louis MO 63121 USA; 5 Maize Genetics Cooperation Stock Center Department of Crop Sciences University of Illinois Urbana IL 61801 USA; 6 Agricultural Research Service United States Department of Agriculture Soil and Nutrition Laboratory Research Unit Cornell University Ithaca NY 14853 USA; 7 Curtis Hall University of Missouri at Columbia Columbia MO 65211 USA; 8 Missouri Botanical Garden 4344-Shaw Boulevard St. Louis MO 63110 USA

## Abstract

The Plant Ontology Consortium (POC) (www.plantontology.org) is a collaborative effort among several plant databases and experts in plant systematics, botany and genomics. A primary goal of the POC is to develop simple yet robust and extensible controlled vocabularies that accurately reflect the biology of plant structures and developmental stages. These provide a network of vocabularies linked by relationships (ontology) to facilitate queries that cut across datasets within a database or between multiple databases. The current version of the ontology integrates diverse vocabularies used to describe *Arabidopsis*, maize and rice (*Oryza* sp.) anatomy, morphology and growth stages. Using the ontology browser, over 3500 gene annotations from three species-specific databases, The Arabidopsis Information Resource (TAIR) for *Arabidopsis*, Gramene for rice and MaizeGDB for maize, can now be queried and retrieved.


**Comparative and Functional Genomics**
*Comp Funct Genom* 2005; **6**: 388–397.
Published online in Wiley InterScience (www.interscience.wiley.com). **DOI:** 10.1002/cfg.496

**Research Article**

### Plant Ontology (PO): a controlled vocabulary of plant structures and growth stages

Pankaj Jaiswal[1#*$], Shulamit Avraham[2], Katica Ilic[3#], Elizabeth A Kellogg[4#], Susan McCouch[1], Anuradha Pujar[1#], Leonore Reiser[3], Seung Y Rhee[3], Martin M. Sachs[5,6], Mary Schaeffer[6,7], Lincoln Stein[2], Peter Stevens[4,8#], Leszek Vincent[7#], Doreen Ware[2,6] and Felipe Zapata[4,8#]

[1] *Department of Plant Breeding, 240 Emerson Hall, Cornell University, Ithaca, NY 14853, USA*
[2] *Cold Spring Harbor Laboratory, 1 Bungtown Road, Cold Spring Harbor, NY 11724, USA*
[3] *Department of Plant Biology, Carnegie Institution of Washington, 260 Panama Street, Stanford, CA 94305, USA*
[4] *Department of Biology, University of Missouri at St. Louis, St. Louis MO 63121, USA*
[5] *Maize Genetics Cooperation, Stock Center, Department of Crop Sciences, University of Illinois, Urbana, IL 61801, USA*
[6] *Agricultural Research Service, United States Department of Agriculture, Soil and Nutrition Laboratory Research Unit, Cornell University, Ithaca, NY 14853, USA*
[7] *Curtis Hall, University of Missouri at Columbia, Columbia, MO 65211, USA*
[8] *Missouri Botanical Garden, 4344-Shaw Boulevard, St. Louis, MO 63110, USA*

**Correspondence to: Pankaj Jaiswal, Department of Plant Breeding, 240 Emerson Hall, Cornell University, Ithaca, NY, 14853, USA. E-mail: pj37@cornell.edu*

*$Authors are listed in alphabetical order except for the first.*

*#These authors contributed equally on the ontology development.*

Received: 19 September 2005
Revised: 21 October 2005
Accepted: 7 November 2005

### Abstract

**The Plant Ontology Consortium (POC) (www.plantontology.org) is a collaborative effort among several plant databases and experts in plant systematics, botany and genomics. A primary goal of the POC is to develop simple yet robust and extensible controlled vocabularies that accurately reflect the biology of plant structures and developmental stages. These provide a network of vocabularies linked by relationships (ontology) to facilitate queries that cut across datasets within a database or between multiple databases. The current version of the ontology integrates diverse vocabularies used to describe *Arabidopsis*, maize and rice (*Oryza* sp.) anatomy, morphology and growth stages. Using the ontology browser, over 3500 gene annotations from three species-specific databases, The Arabidopsis Information Resource (TAIR) for *Arabidopsis*, Gramene for rice and MaizeGDB for maize, can now be queried and retrieved. Copyright © 2006 John Wiley & Sons, Ltd.**

**Keywords:** plant growth stage; controlled vocabulary; plant ontology; plant development; plant anatomy; plant morphology; gene expression; phenotype

### Introduction

The amount of information about genes and gene functions in flowering plants has increased dramatically with the full genome sequencing of *Arabidopsis* 1 and rice (*Oryza*)6 and from the emerging sequences of *Populus, Medicago*, maize (*Zea mays*), and tomato (*Solanum lycopersicum*). This makes it increasingly important to describe and classify gene expression and phenotype information in ways that allow easy comparison. Biologists want to be able to use the information stored in disparate databases to ask biologically interesting questions. They want to know, for example, which genes or gene products contribute to the formation and development of the endosperm, what agronomically important genes contribute to delayed or early flowering, or what evidence there is that the *Arabidopsis Gibberellin 3 β-hydroxylase (GA4)* gene is expressed at the time of seed germination. Often researchers want to find gene products that have similar functions, phenotypes or expression profiles in different plants. To support this kind of research, databases organize and annotate information using controlled vocabularies that standardize descriptions of biological entities,

Copyright © 2006 John Wiley & Sons, Ltd.

such as genes, gene products, source materials used for EST libraries, microarray and proteomics experiments and phenotypes, in ways that enable a computer to search for and identify common features of all these data types in different species.

The Plant Ontology Consortium (POC: **http://www.plantontology.org**) was formed to develop controlled vocabularies that could be used to generically describe plant anatomy, growth and developmental stages and to use those vocabularies to annotate various datasets from plant genomics and genetics projects11. The goal of the project is to construct a set of clearly defined vocabularies that can be used to construct queries in Gramene15,16, TAIR12 and MaizeGDB8, using a single common set of terms. It soon became apparent that such a set of terms would be a useful resource for the entire plant science community.

We initiated our work by integrating basic terms from three species-specific ontologies that had previously been developed for rice, maize and *Arabidopsis* by the Gramene15, MaizeGDB14 and TAIR3,12 databases, respectively. We are now extending this initial phase of work and introducing terms required by the families Solanaceae and Fabaceae. The vocabularies we have developed represent common concepts in plant biology and offer a unifying language that serves as a foundation for describing spatial and temporal aspects of flowering plant biology in a comparative context.

The use of ontological methods to organize biological knowledge is an active area of research and development2,10. The POC endeavours to develop cross-species vocabularies that can be used by plant databases and researchers to annotate data objects such as genes, gene products and phenotypes in a consistent way. The two ontologies under development are plant structure (anatomy, including morphology) and growth and development (temporal). This paper introduces the two ontologies and describes ways in which they are used and made available.

### The Ontologies

The plant structure ontology is a controlled vocabulary of botanical terms describing the morphological and anatomical structures of plants. These structures include organs, tissues and cell types and relationships among them. Examples are 'stamen', 'ovule', 'petal', 'parenchyma', 'guard cell', etc. The structure ontology does not include attributes (or characters) of the structural components, e.g. the term 'ovary' is included but whether the ovary is superior and inferior is not described. The structure ontology also does not include subcellular structures, which are covered by the cellular component domain of the Gene Ontology (GO)5.

The plant growth and development (temporal) ontology describes morphological and anatomical landmarks that define various stages of a plant's life cycle (growth stages) and plant structures (developmental stages). Examples are 'vegetative stage', 'reproductive stage', 'germination', etc. Detailed descriptions of the ontologies and their organizing principles will be presented elsewhere.

PO is the only controlled vocabulary that is available in the ontology format to the flowering plant researchers and databases for use in the annotation of gene and phenotype expression datasets. Unlike the many plant vocabularies and glossaries that are organized either as a list of terms or sometimes in a simple hierarchy, where specific terms are structured as children of more general (parent) terms (Figure 1a), the PO terms are organized hierarchically, such that one child can have more than one parent (Figure 1b). For example, the plant structure term 'trichoblast', has two parents, 'cell' and 'root epidermis'. This is because a 'trichoblast' is a type of 'cell', and is part of 'root epidermis'. Compared to the simple hierarchies described in Figure 1a, where there are no defined relationships among terms, in the PO three types of relationships were introduced to link a child term to a parent term (Figure 1b), thus creating a network of botanical terms depicting the morphological and developmental complexity of plants. The relationships are: (1) 'part of', indicating a composition or constituency relationship, e.g. 'root hair' is a part of 'root epidermis'; (2) 'is a', indicating a generalization relationship where a child term is a type of a parent term; e.g. 'root hair' is a 'cell'; and (3) 'develops from', indicating a derivation relationship where a child is derived from the parent; e.g. 'root hair' develops from 'trichoblast'. Thus, when any gene is annotated as expressed in a trichoblast, e.g. *Arabidopsis thaliana* gene *ADL1C*, a member of the 68 kDa dynamin-like protein family7, it is automatically associated with both of the parent terms, 'cell' and 'root epidermis'

Copyright © 2006 John Wiley & Sons, Ltd.

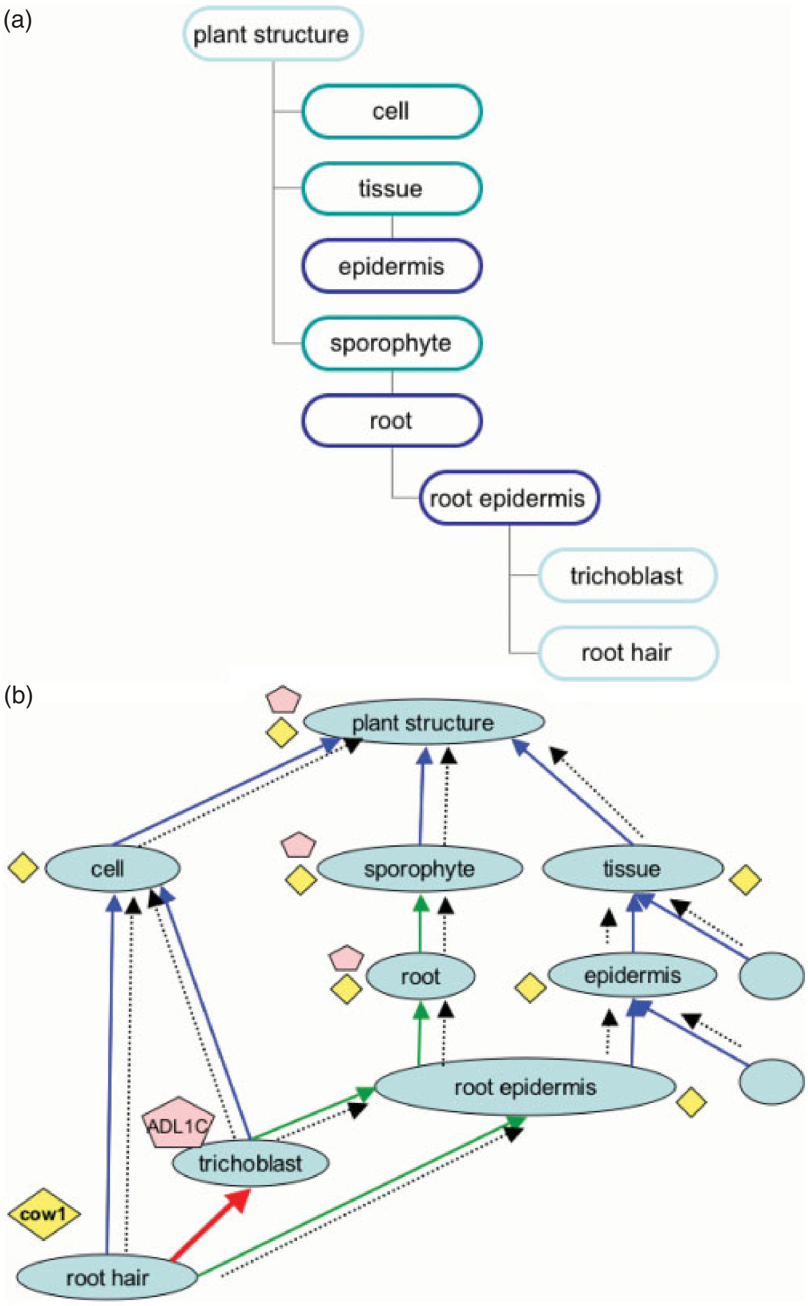

**Figure 1.** (a) The parent and child term organization in a simple hierarchy. Such a tree often allows only one parent–child relationship and lacks relationship types. (b) The parent and child term organization in the plant ontologies. In this example, a 'trichoblast' is a type of 'cell' and 'cell' is a 'plant structure'. Similarly, 'root hair' is a type of 'cell', is part of 'root epidermis' and develops from 'trichoblast'. The blue lines are for 'is a', green for 'part of' and red for 'develops from' relationships. Arrows indicate a directional path; those with dotted lines depict the directional path along which the associations are accumulated by parent(s) from the children terms. The diamond- and pentagon-shaped icons depict the cell type-specific expression based association of *cow1* and *ADL1C Arabidopsis* genes, respectively. The associations are not propagated to parent terms if the 'develops from' relationship is encountered, e.g. '*cow1*' does not show up with 'trichoblast' when the associations are acquired from children terms. However, the associations acquired by the parent terms are allowed if the relationship with their children terms are 'is a' and 'part of', e.g. both '*cow1*' and '*ADL1C*' show up with parent terms such as 'root epidermis' and 'cell'. In this figure not all the parent terms of a child are shown

Copyright © 2006 John Wiley & Sons, Ltd. 

(Figure 1b). A query to retrieve all genes expressed in 'root epidermis' will then automatically retrieve gene annotations to 'trichoblast' without having to annotate the gene explicitly to 'root epidermis'; the connection between the more specific (child) term and more general (parent) term is produced automatically by the ontology. However, annotations do not propagate upwards through the hierarchy if the 'develops from' relationship is encountered, because the derivation does not assure that the gene expressed in a child term is also expressed in the parent structure. For example, *Arabidopsis thaliana* gene *can of worms1* (*cow1*)4, a phosphatidylinositol transfer protein essential for root hair tip growth that is annotated to the term 'root hair', can be retrieved by its parents, 'cell' and 'root epidermis', but not by 'trichoblast' (Figure 1b) because it can be said to be expressed in the cell and in root epidermis if it is expressed in a type of cell or a part of root epidermis. However, this gene cannot be said to be expressed in the trichoblast because the expression in a derived cell does not guarantee its expression in the progenitor cell. This allows the user to query the associations to find all the genes that are expressed in 'root hair' but not in 'trichoblast', or find all the genes that are expressed in 'root epidermis' but not in 'trichoblast'. However, this will require the researchers and database curators to annotate the genes and other data objects appropriately, by making sure that the association is specific to the derived structure only and provide additional annotation if it is true for the progenitor structure as well. The design of the ontologies also follows a True Path Rule (TPR). According to this, the pathway from a child term to its top-level parent(s) must always be true.

Every term in the plant structure and growth and development ontologies is identified by a unique identifier or Accession No. All terms in the PO are associated with a definition that concisely describes the meaning and context of the term and is linked to source reference(s). Whenever possible, we use internationally accepted definitions obtained from plant biology text books, journal articles and other expert sources. When a published definition is unavailable, one is written by the PO curators.

Most terms in the PO have synonym(s), which indicate alternative names. The extensive use of synonyms is critical for cross-species ontology, because the same botanical structure is often called by a different name in different species. For example, the inflorescence in rice (*Oryza*) is called a 'panicle', whereas it is called a 'cob' and 'spike' in sorghum and in Triticeae (wheat, oat and barley), respectively. Synonyms may have their own references.

In some cases the *sensu* (in the sense of) qualifier is used along with the taxonomic name to make the application of the term more precise. A good example of this is the incomplete flower of Poaceae, which is called a 'floret'. Similar structures in the Asteraceae that are called florets are quite different in organization and it would be a mistake to conflate them; therefore it is best to create two terms, 'floret *sensu* Poaceae' and 'floret *sensu* Asteraceae'. The standard format for this is 'term (*sensu* taxon)', e.g. floret (*sensu* Poaceae) and floret (*sensu* Asteraceae).

### Comparison to other ontologies

A number of anatomy and temporal ontologies are available on the OBO website10. Except for PO and GO, the majority of these ontologies are designed specifically for one organism. All of the anatomy and temporal ontologies use the same relationship types as PO ('is a', 'part of', and 'develops from'). Also, synonymy is used in all ontologies. PO is designed to cover the plant structures and growth stages of multiple species representing the taxon Angiosperm (flowering plants) and GO5 is designed to describe molecular function, biological process and cellular component of all species. In GO, there are cases where a term may have different meanings when applied to different organisms. For example, 'gametogenesis' in plants is very different from 'gametogenesis' in metazoans. Such terms are distinguished from one another by their human readable definitions and by the *sensu* designation in the term name, as in the term 'gametogenesis (*sensu* Magnoliophyta)' and 'gametogenesis (*sensu* Metazoa)'. In PO, the *sensu* designations are currently being used to distinguish between two homologous plant structures with the same name, which are morphologically distinct and have different component parts (children terms) in different taxons. In addition to the *sensu* terms, the PO also has taxon-specific terms that do not require the *sensu* designation. Such terms are often part of a sub-tree that is generated by adding children

Copyright © 2006 John Wiley & Sons, Ltd.

terms as subtypes of parent terms, e.g. ‘tassel’, the male inflorescence in maize (*Zea mays)*, is a subtype of a generic ‘inflorescence’ term because it is functionally and structurally monoecious (imperfect unisexual flowers present on the same plant). Although ‘tassel’ is specific for *Zea mays*, it does not appear anywhere in the ontology as either a homologous, analogous or a generic plant structure term, therefore the term ‘tassel (*sensu* Zea mays)’ is not required.

### Maintenance and consistency checks

Changes to the ontologies, such as addition and modification of terms, need to be approved by the ontology curators prior to committing them to the ontology files and the database. During this process, a term may be removed from the active ontology but it is not deleted from the ontology files; rather, it is tagged ‘obsolete’. A term is not made obsolete if a change in the term name or its definition does not alter the meaning of the term. When a term name or the definition changes significantly, such that it alters the meaning of the term, it is assigned a new PO ID and the old ID is considered obsolete. In the ontology browser, an obsoleted term becomes a child of the obsolete node. Obsolete terms are identified in the OBO format flat file by the ‘is_obsolete: true’ tag12. In addition, when a term is marked obsolete, the word ‘OBSOLETE.’ is inserted at the beginning of the term definition and a comment is added to explain why the term has become obsolete. It may also suggest alternative terms to use for annotation.

Each term in the PO is a unique subtype of either the plant structure or the plant growth and development ontologies, and terms are not shared between the two ontologies. Each term must have a parent with ‘is a’ relationship. This rule mandates that, for example, every term in the plant structure ontology ‘is a’ plant structure. For consistency and integrity checks, we use the ‘Obol’ tool9, which is designed to search for missing ‘is a’ relationships and suggest the putative parent terms with ‘is a’ relationships. Upon finding such inconsistencies in the existing ontologies, the group validates the results, and the terms are then assigned appropriate parent terms with ‘is a’ relationships. More information on the Obol results is available at **http://www.fruitfly.org/~cjm/obol/pong/**.

### POC Resources

### Ontology browser

The Plant Ontology browser is available at **http://www.plantontology.org/amigo/go.cgi**. This is a web-based tool for searching and browsing ontologies and their associations to data. It has been developed by the GO consortium (**http://www.geneontology.org/GO.tools.shtml#in_house**) and modified to suit our needs. To browse, clicking on the [+] sign in front of the term expands the tree to show children terms (Figure 2). This view provides information on the PO ID of the term, term name, followed by a number of associated data, such as genes. For every green-coloured parent term, a summary of the data associated to its children terms is presented as a pie chart. The user has an option to filter the number of associated data displayed, based on species, data sources and evidence codes. The icons for [i], [p] and [d] suggest the relationship types between the parent and child term, as described in the legend. While browsing, a user can click on the term name to get the details at any time (Figure 3b)

In addition to the browse utility, users may search by entering the name of a term or a gene, e.g. querying with ‘gametophyte’ results in seven terms, of which three are from the plant structure ontology and four are from the growth and development ontology. To avoid getting a large list, users may choose the ‘exact match’ option before submitting the query. A search for ‘gametophyte’ choosing ‘exact match’ gives one result (Figure 3a). A user may browse the parents and children of this term by clicking on the blue-coloured tree icon and following the [+] sign next to the term name, which suggests that there are additional terms under this term (Figure 3b), or simply clicking on the term name ‘gametophyte’ for more details. The term detail page provides information on the ID, aspect (plant structure or growth and development), synonyms (if any), definition, external references (if any) and the associated data. The association section allows a user to select the annotation source, species and the evidence code used to make the annotation to limit the data displayed. The list of associated data

Copyright © 2006 John Wiley & Sons, Ltd.

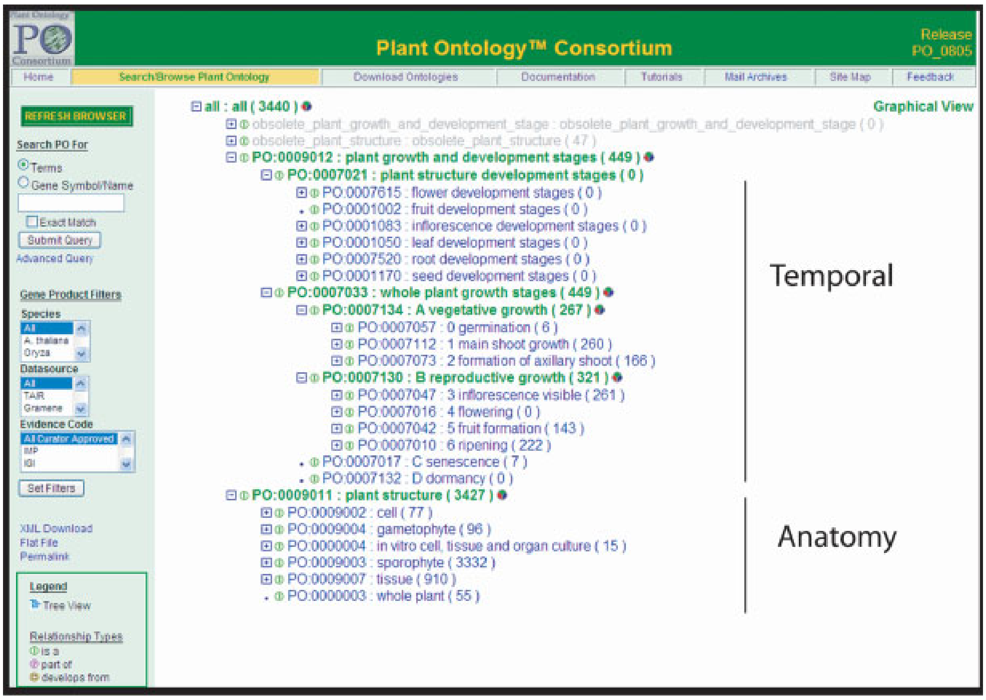

**Figure 2.** A view of the ontologies using the plant ontology (PO) browser. Users can go to the website (**www.plantontology.org**) and click on 'search/browse plant ontology' from the navigation bar menu. On the ontology browser page, the two options are to search or browse. For searching, type the term name, e.g. 'gametophyte', and select the 'term' option or type the gene name, e.g. 'Du8', and select the 'gene symbol/name' option before clicking the submit button. For a gene search, there are additional 'gene product filters' options to chose from. If you are browsing, simply click on the [+] icon before the term name, which will expand the tree by opening the children terms. The PO ID is the term's Accession No., and the number followed by the term name is the total number of associations to the genes a term has; this number will change depending on the gene product filter a user may have chosen. Users can also get a pie chart showing the distribution of data associations to a term's children term. In this figure, the general level (top level) terms in the plant structure and growth and development ontologies are displayed using the browse option gives information about the name, symbol, type (e.g. gene), the annotation source and the species, in addition to the evidence used for making the association to the term. The gene symbol provides a hyperlink to the gene detail page, and the data source links to the same entry on the provider's website. This allows a user to search for extended details that may not be provided in the POC database, such as information on genome location, biochemical characterization, etc. The evidence code, such as inferred by mutant phenotype (IMP) or inferred by direct analyses (IDA), links to the citation used in inferring the gene's association to the plant ontology term. A complete list of evidence codes with a list of experiment types can be found at **http://www.plantontology.org/docs/otherdocs/evidence_codes.html**. For help at any time, users can click on the 'help' menu at the bottom of the browser page or visit the link, **http://www.plantontology.org/amigo/docs/user_guide/index.html**.

### Tutorials

We encourage the use of plant ontologies by both the plant databases and individual researchers. A set of tutorials can be viewed or downloaded from **http://www.plantontology.org/docs/otherdocs/tutorials.html**. These tutorials include a quick tour of the PO website and how to use the ontology browser to search and browse the ontologies and associated data.

### Downloads

The vocabularies, annotations, mappings and the database are in the public domain and are readily accessible via instructions provided on the

Copyright © 2006 John Wiley & Sons, Ltd. 

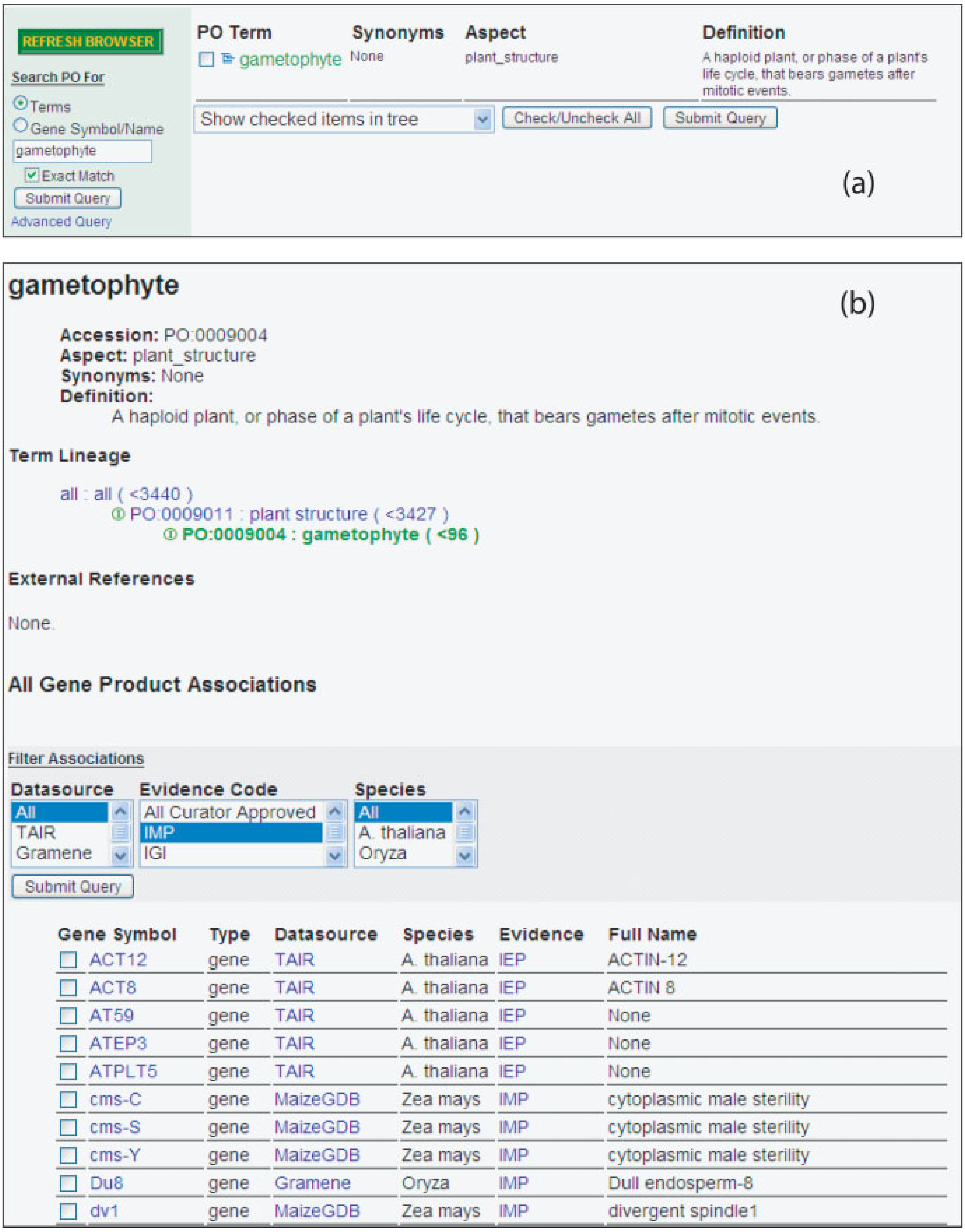

**Figure 3.** The ontology search results and the term detail page. (a) The search results view. Searching with the term 'gametophyte' using the exact option returns a single term. In order to browse this term's lineage in the ontology, simply click on the blue-coloured tree icon next to the term name. For more details on the term, click on the term name. (b) The ontology term detail page. This provides information on the ontology term, including Accession No., definition and, if available, synonyms, comments and data associations. The display of associated data is configurable by users

Copyright © 2006 John Wiley & Sons, Ltd. 

download page (**http://www.plantontology.org/download/download.html**).

The ontology database and data access software have been developed by the Gene Ontology Consortium (**http://www.godatabase.org/dev/database/**) and consist of a MySQL database of ontologies and associations, a Perl object model and Application Programmer Interface (API) to simplify database access. The database is released monthly. The schema represents generic graphs, including the PO structure (a directed acyclic graph, or DAG) relationally. At the core of the schema are two relational tables for capturing all terms and relationships between the terms. The full version of the ontology database in MySQL described above can be downloaded for local use. The POC ontologies are also available in OBO (Open Biological Ontology) flat file formats13, which are frequently updated.

### Mappings to other vocabularies

Individual databases wishing to use the PO may choose to retire their own plant structure and growth stage vocabularies and convert to using the PO. To help such users perform the conversion, the development and use of mapping files is recommended to help with the transition. Species-specific databases may wish to adopt a dual option during a transitional period, whereby they provide side-by-side PO and species-specific vocabularies to encourage users to familiarize themselves with the new terms. More information about the usage and format of mapping files are found online (**http://www.geneontology.org/GO.format.shtml#mappings**).

### Data associations

Member databases of the POC submit data associations to PO terms to the POC website. This allows researchers to quickly and easily obtain *Arabidopsis*, rice, and maize genes and other data objects associated to terms on the basis of the gene expression patterns or phenotypes. An example (**http://www.plantontology.org/amigo/go.cgi?view=details&show_associations=terms&search_constraint=terms&depth=0&query=PO:0009046**), shows the genes from *Arabidopsis*, rice, and maize that are annotated to 'flower' (PO:0009046). Hypertext links allow researchers to browse the source databases to obtain additional information about the listed genes. Presenting gene associations on the POC website demonstrates the utility of the ontologies, and also provides a valuable tool for researchers performing comparative genomics. As circumstances warrant, we will add other types of associations, such as microarray and proteomics results, mutant stocks and QTL phenotypes, and gene associations from other databases and organisms. We encourage submission of data associations to PO terms from any interested database or researcher. A suggested association file format that can be accessed at **http://www.plantontology.org/docs/otherdocs/assoc-file-format.html**. This includes information on the source, ID, object type (e.g. gene), references or citations, and evidence used for making the association.

### Collaborations and community inputs

We wish to increase membership and extend the usage of PO to the broader plant community. Already, a large number of collaborators have shown interest or have already started using the PO to annotate their datasets, as listed in Table 1. With recent initiatives on creating genomic and functional resources for several other species (Table 1), we anticipate that the current set of vocabularies will require the addition of new terms, synonyms and definitions as well as modifications to the structure. Accomplishing this requires consultation with anatomy and development experts and different research user communities. We encourage databases and individual researchers to contact us if they are suggesting new terms, modification of existing definition(s), term-to-term relationships or even interested in joining the POC by sending an e-mail to: **po-dev@plantontology.org**. More information about joining POC can be found online: **http://www.plantontology.org/docs/otherdocs/charter.html**.

### Future directions

Our current and near-future development efforts are focused on the introduction of species-specific terms into the PO to accommodate annotations from legumes (*Medicago* and soybean), Solanaceae (tomato), poplar (*Populus*) and Triticeae (wheat,

Copyright © 2006 John Wiley & Sons, Ltd. 

**Table 1.** Collaborations and users

| Resources | Description and web link |
|---|---|
| ABRC | Stock centre for seeds and DNA resources; **http://www.biosci.ohio-state.edu/~plantbio/Facilities/abrc/abrchome.htm** |
| ArMet | A resource for *Arabidopsis*/plant metabolomics; **http://www.armet.org/** |
| Barleybase | Repository for barley microarray data; **http://www.barleybase.org** |
| GARNet (Genomic *Arabidopsis* Resource Network) | Analysis support system for genomics and metabolomics in *Arabidopsis*; **http://garnet.arabidopsis.org.uk** |
| Generation Challenge Program | Program on genetic resources, genomic and crop information systems; **http://www.cgiar.org/pdf/cpunlocking.pdf; http://www.generationcp.org** |
| Genevestigator | *Arabidopsis* Microarray Database and Analysis Toolbox; **https://www.genevestigator.ethz.ch/** |
| GrainGenes | The genome database for small-grain crops (wheat, oat and barley); **http://www.graingenes.org** |
| Gramene | A resource for comparative grass genomics and genetics; **http://www.gramene.org** |
| IR64 Mutant Database | A collection of rice mutant stocks; **http://www.iris.irri.org:8080/IRFGC/ir64.shtml** |
| MaizeGDB | A resource for maize Genetics/Genomics information; **http://www.maizegdb.org** |
| Medicago truncatula | A resource for *Medicago* Genetics/Genomics information; **http://www.medicago.org** |
| Monsanto Co. | Genome Knowledge Enhancement Program (GKEP), agriculture biotechnology and crop improvement; **http://www.monsanto.com** |
| NASC (Nottingham *Arabidopsis* Stock Center) | Seed stock centre and information resources to the International *Arabidopsis* Genome Program; **http://arabidopsis.info** |
| Oryzabase | A resource for rice genetic, genomic and phenotypic data; **http://www.shigen.nig.ac.jp/rice/oryzabase/** |
| Pioneer Hi-Bred International Inc. | Agriculture biotechnology and crop improvement; **http://www.pioneer.com/** |
| PlaNet | A network of plant genome database for the systematic exploration of the genes and their function; **http://www.eu-plant-genome.net/** |
| Plant MIAME | Minimum Information About a Microarray Experiment—MIAME for Plant Genomics; **http://arabidopsis.info/info/MIAME-plant_whitepaper_sept23_2004.pdf** |
| PLEXdb (Plant Expression Database) | Unified public resource for large-scale plant gene expression; **http://www.plexdb.org/** |
| Rice Oligonucleotide Array Project | Rice microarrays; **www.ricearray.org** |
| SGN (SOL Genomics Network) | A resource for biology of the family Solanaceae; **http://sgn.cornell.edu** |
| Soybase | A resource for soybean genetics/genomics information; **http://soybase.agron.iastate.edu/** |
| TAIR (The *Arabidopsis* Information Resource) | A resource for Arabidopsis genomics and genetics information; **http://www.arabidopsis.org** |
| WAtDB (Wageningen *Arabidopsis thaliana* Database) | A resource for *Arabidopsis* mutants, transgenics or natural variants; **http://www.watdb.nl** |

oat and barley). We expect PO development to be an ongoing activity as we seek to create a robust resource which is responsive to new discoveries that change in our understanding of plant biology.

### Acknowledgements

We gratefully acknowledge the Gene Ontology Consortium for making the ontology browser (AmiGO), editor (DAG-Edit), Obol tool and database software available and for help with modifications and implementation. We are grateful to many researchers, reviewers, database groups and curators for help in reviewing, adding new terms to the ontology and using them in their annotation work. The extensive list of people we wish to acknowledge is available online at **http://www.plantontology.org/docs/otherdocs/collab.html** and **http://www.plantontology.org/docs/otherdocs/acknowledgment_list.html**. This work is supported by the National Science Foundation award (Grant No. 0321666) to the Plant Ontology Consortium and USDA-ARS, USA.

### References

1. Arabidopsis Genome Initiative. 2000. Analysis of the genome sequence of the flowering plant *Arabidopsis thaliana*. *Nature* **408**: 796–815.

Copyright © 2006 John Wiley & Sons, Ltd. 

2. Bard J, Rhee SY. 2004. Ontologies in biology: design, applications and future challenges. *Nature Rev Genet* **5**: 213–222.
3. Berardini TZ, Mundodi S, Reiser L, *et al*. 2004. Functional annotation of the *Arabidopsis* genome using controlled vocabularies. *Plant Physiol* **135**: 745–755.
4. Bohme K, Li Y, Charlot F, *et al*. 2004. The *Arabidopsis COW1* gene encodes a phosphatidylinositol transfer protein essential for root hair tip growth. *Plant J* **40**: 686–698.
5. Clark JI, Brooksbank C, Lomax J. 2005. It's all GO for plant scientists. *Plant Physiol* **138**: 1268–1279.
6. International Rice Genome Sequencing Project. 2005. The map-based sequence of the rice genome. *Nature* **436**: 793–800.
7. Kang B, Rancour DM, Bednarek SY. 2003. The dynamin-like protein ADL1C is essential for plasma membrane maintenance during pollen maturation. *Plant J* **35**: 1–15.
8. Lawrence CJ, Dong Q, Polacco ML, Seigfried TE, Brendel V. 2004. MaizeGDB, the community database for maize genetics and genomics. *Nucleic Acids Res* **32**: D393–397.
9. Mungall CJ. 2004. Obol: integrating language and meaning in bio-ontologies. *Comp Funct Genom* **5**: 509–520.
10. OBO. 2005. Open Biological Ontologies; **http://obo.sourceforge.net/**
11. Plant Ontology Consortium. 2002. The Plant Ontology™ Consortium and plant ontologies. *Comp Funct Genom* **3**: 137–142.
12. Rhee SY, Beavis W, Berardini TZ, *et al*. 2003. The *Arabidopsis* Information Resource (TAIR): a model organism database providing a centralized, curated gateway to *Arabidopsis* biology, research materials and community. *Nucleic Acids Res* **31**: 224–228.
13. The Gene Ontology database. 2005. The Open Biological Ontology (OBO) Flat File Format; **http://www.geneontology.org/GO.format.shtml#oboflat**
14. Vincent PL, Coe EH, Polacco ML. 2003. *Zea mays* ontology — a database of international terms. *Trends Plant Sci* **8**: 517–520.
15. Ware DH, Jaiswal P, Ni J, *et al*. 2002. Gramene, a tool for grass genomics. *Plant Physiol* **130**: 1606–1613.
16. Yamazaki Y, Jaiswal P. 2005. Biological ontologies in rice databases. An introduction to the activities in Gramene and Oryzabase. *Plant Cell Physiol* **46**: 63–68.

Copyright © 2006 John Wiley & Sons, Ltd. 
